# Retraction: Curcumin Suppresses Proliferation and Migration of MDA-MB-231 Breast Cancer Cells through Autophagy-Dependent Akt Degradation

**DOI:** 10.1371/journal.pone.0283354

**Published:** 2023-03-15

**Authors:** The PLOS ONE Editors

Following the publication of this article [1], concerns were raised regarding results presented in Figs 3, 5, 7, 8, and S3. Specifically, the following panels appear similar:

- Lanes 2–4 of the Fig 5C Atg5 panel and the Fig 8C Akt panel.
- Lanes 2–5 of the Fig 3A p62 panel of this study [1] and the Fig 4A Ac-STAT3 panel of [2] when flipped horizontally.
- The Fig 3D Tubulin panel of this study [1] and the Fig 4A STAT3 panel of [2].
- The Fig5C Akt panel of this study [1] and lanes 2–5 of the Fig 7A p-STAT3 panel of [3].
- Lanes 1–2 of the Fig 5C Akt panel of this study [1] and lanes 1–2 of the Fig 4C Adpn panel of [4].
- The Fig 7C Akt panel of this study [1] and the Fig 4G PEPCK panel of [2].
- The Fig 8C p62 panel of this study [1] and lanes 2–4 of the Fig 6B HpG2 Lysate p-STAT3 panel of [3].
- Lanes 1–4 of the Fig S3A AMPK panel of this study [1] and the Fig 2D IRE1α panel of [5].
- Lanes 2–5 of the Fig S3A AMPK panel of this study [1] and the Fig 2D p-PERK panel of [5].

The corresponding author commented that some western blot images were misused in this article [1] and requested the retraction of the article.

In light of the concerns affecting multiple figure panels that question the integrity of these data, the *PLOS ONE* Editors retract this article.

CW agreed with the retraction. FG, YD, YZhang, YZhou, and ML either did not respond directly or could not be reached.
